# Field-friendly MUNNG^®^ optima simple test kit for quick qualitative assessment of iodine and iron presence in double-fortified salt

**DOI:** 10.3389/fnut.2023.1059332

**Published:** 2023-01-26

**Authors:** Ujwala Godbole, Divya Gupta, Nachiket Godbole, Madan Godbole

**Affiliations:** ^1^Institute of Bioscience and Technology, Shri Ramswaroop Memorial University, Barabanki, India; ^2^Department of Pharmaceutical Sciences, University of Oklahoma Health Sciences Centre, Oklahoma City, OK, United States; ^3^Food and Micronutrient Analysis Laboratory, KLE University, Belagavi, Karnataka, India

**Keywords:** field friendly, rapid testing kit, iodine, iron, detection, double fortified salt

## Abstract

**Background:**

Data from several efficacy studies and a long-term effectiveness study have encouraged the governments to adopt a policy of providing double-fortified salt (DFS) in the Mid-Day Meal (MDM) programs in government schools across India. These envisaged food security events are likely to boost the manufacturing of DFS in a big way. Thus, it becomes pertinent to come up with a robust monitoring system involving community and field workers for quality checks. It is imperative to equip these field workers with simple testing kits (STKs) capable of qualitative detection of iron and iodine in DFS. As the consumer acceptance of foods is based on several factors including sensory characteristics, performance, convenience, cost, nutrition, and product image, a variety of iron compounds are in use for fortification. However, it becomes challenging to provide a kit that can overcome the chemical masking of iodine detection by iron compounds.

**Objectives:**

We aimed at (1) the development of a field-friendly STK for quick qualitative assessment of iodine and various forms of iron present in DFS, (2) to check its validity under field conditions.

**Methods:**

We put in place reagents combined using known chemical reactions and balanced use of oxidants to overcome the problems of encapsulation and to maximize the use, by enabling reagent combination to react with all forms of iron.

**Results:**

The kit reagents successfully detect iodine as well as three commonly used iron fortificants in DFS. Published field trials confirmed the specificity and sensitivity of the developed kit. The simplicity and use of the kit by a field worker can be seen in the enclosed video.

**Conclusion:**

The combination of improvised kit reagents allows early detection of iron and iodine in DFS. Iron is detected in a variety of iron-containing fortifications. The provision of diluted H_2_O_2_ ensures the presence of oxygen-free radicals that enhances iodine release captured by concentrated KI making iodine detection an easy task.

## Introduction

The success of the Universal Salt Iodization (USI) program in India is ascribed to the efforts of the Indian Union Government to pass a law to ban the supply of non-iodized edible salt since 1986, and the vigorous perusal of the policy by state governments at ground level. Recent surveys estimate the use of iodized salt by 96.5% and 91.5% of the households in urban–rural areas, respectively, and those consuming adequately iodized salt (15 ppm) at 76% (1, 2). The setting up of a successful monitoring mechanism in place was achieved through the availability of a simple but effective salt iodine testing kit (STK).

The considerable success of USI has also led to efforts to use salt as a vehicle for making available other essential minerals such as iron (double fortified or DFS) to alleviate the alarmingly high prevalence of anemia in women of reproductive age (WRA) and children from the vulnerable groups among the population. Over the last 20 years, the National Institute of Nutrition (NIN), Hyderabad, and the University of Toronto have contributed significantly in the former carrying out efficacy studies (3–10) and the latter providing technologies for the manufacturing of DFS.

The failure to upscale DFS production and its acceptability by masses may be attributed to the failure in demonstrating the significant improvement in circulating levels of hemoglobin through large-scale field trials that could prove the effectiveness of DFS distribution through the public distribution system. This would also explain the relevance of DFS as a public health improvement measure (11–13). In contrast, the reduction in mild anemia is 6 percentage points or approximately 30%; the point estimate for moderate or severe anemia is also quite large and negative (13 percentage points) but the effects are not statistically significant. Some of the prior studies reported a similar effect size, found a reduction in anemia by 20% in India, and reported an effect size of 34.5% in Peru, while some studies were unable to find any significant impacts of DFS on anemia in India (13, 14). The issue now seems to have settled in favor of the distribution of DFS in MDMs and the Integrated Child Development Scheme (ICDS). This was supported by experimental evidence on child health and human capital outcomes from the longer-term follow-up of a school-based nutrition intervention in India (15). Using panel data, the effectiveness of using iron- and iodine-fortified salt in school lunch meals to reduce anemia among school children was examined. After 4 years of treatment, the treated children, on average, showed higher hemoglobin levels and a lower likelihood of anemia relative to the control group (15, 16). Interestingly, the intervention did not have an impact on the cognitive and educational outcomes (15, 16). Realizing the impact, long-term follow-up of early childhood health interventions is important for human capital accumulation. The governments of Gujarat and Madhya Pradesh have taken upon themselves a commitment to provide DFS through MDM. The estimated consumption of 1,17,546 and 1,80,000 metric tons of DFS, in the year 2018, through PDS in seven states (17, 18) is likely to increase several folds after the state governments take cognizance of the positives reported in recent literature and the Prime Minister speaking in favor of micronutrients to be included in both MDM and ICDS. As the scaling up of DFS production is likely to occur from the present manufacturers that produce 8000 metric tons of premix adequate for around 10,00,000 metric tons of DFS production, it raises several questions about quality control. Given the installed capacity of 9,14,000 metric tons per annum, it will not be difficult for India to fulfill the upscaling demand. Four factors that are key to ensuring the quality production of DFS include: (1) the quality of iron compound used, (2) the amounts of iodine and iron that are required, (3) the stipulated quality of salt needed, and 4) the in-country ability to test, enforce, and/or otherwise ensure a quality DFS formulation and end product. In the Indian context, the first three quality parameters have been fixed by the Food Safety and Standards Authority of India (FSSAI) and the Bureau of Indian Standards (BIS) (18). Currently, four formulations are in use, namely, (i) *Types 1b/1c* containing encapsulated ferrous fumarate (EFF), 1c produced with additional compounds like color stabilizer [sodium hexametaphosphate (SHMP)] and a color masking agent [titanium dioxide (TiO2)], and former, i.e., 1b in which EFF is extruded with a binding agent [hydroxy propyl methyl cellulose (HPMC)]. On the other hand, *Type 2* contains ferrous sulfate (FS) heap-hydrate along with color stabilizer, and Type 3, in addition to the above, contains other micronutrients such as folic acid, zinc, vitamin A, and vitamin B-12 as well as ascorbic acid. Others like Type 4 containing EDTA salt and Type 5 containing micronized ferric pyrophosphate (MFPP) are also in use in some countries. Indian manufacturers mainly use *Types 1b/1c type premix* and are phasing out the use of *Type 2 premix.*

However, quality control/assurance measures necessary to maintain a consistent quality of DFS production throughout the supply chain, down to PDS shops and consumer end, are still not established. The reasons include (a) the requirement of a laboratory, (b) the high cost of staff training, (c) the use of different iron formulations (premixes) prescribed in different countries for salt fortification, and (d) different methodological requirements. These hurdles necessitate sending samples abroad for analysis to specialized laboratories which makes it an impracticable preposition.

In the past, two attempts have been made to validate the iron and iodine levels in DFS (19–22). The efforts to develop iron and iodine analytical methods, though based on known chemical reactions, did not find acceptance by community workers and field staff (19–22), and despite a Supplementary video depicting the performance of the methodology (22), its utility has not gained wide acceptability.

We, therefore, embarked on the development of a field-friendly kit that can simultaneously detect both iron and iodine in DFS samples, modifying the known chemical reactions by the inclusion of a eutectic mix of H_2_O_2_ and water, to ensure the release of iron from its chemical as well as encapsulated form, and overcome the masking effect of iron compounds on iodine starch reaction.

## Materials and methods

### MUNNG^®^ optima

We recently developed and validated a kit useful for field-level monitoring. Analytical grade RANKEM brand chemicals were purchased from Science World No: 31, Shivanna Layout, H.V. Halli, Rajarajeshwari Nagar, Bangalore, India-560 098. The differential solubility of iodine in an acidic KI solution allows its detection with a starch reagent, resulting in the development of a violet-blue color even in the presence of a large amount of iron (23). To ensure ease of use for field workers and cost-effectiveness, we improvised and validated the protocol. The development of two different color endpoints is not only suitable for the identification of iodine but also many compounds used for iron fortification including the encapsulated forms. MUNNG^®^ is a registered trademark in the name of the first author-run manufacturing unit.

*Principle:* The optima kit follows the basic principle of the release of iron–thiocyanate and iodine–starch interaction under acidic conditions (23). The kit is formulated based on the reaction of ferrous iron and peroxides ii reactions with hydrogen peroxide, in the presence/absence of oxygen (24–26).

*The stoichiometry of iodine:* Starch reaction generates many folds of iodine. The free iodine first reacts with excess iodide anions to ultimately form penta-iodide anion that reacts with starch to form a deep-blue complex. The depth and intensity of the blue color are directly related to the presence of excess iodide anions. The generation of high amounts of iodide anions is ensured by several folds of the amount of iron fortificants present in DFS compared to iodine. This condition is met through the use of concentrated KI.


$$H_{2}\,O_{2}\;I_{2}\,\left(\mathrm{Conc}\,\mathrm{KI}\,\mathrm{captures}\,\mathrm{released}\,\mathrm{iodine}\right)$$

-⁣-⁣↓⁣-⁣-⁣-⁣-⁣↓⁣↑⁣-⁣-

$$\overset{-}{I}\overset{+H^{-},I_{2}}{⟶}I_{2}$$

$$F\,e^{2+}\to F\,e^{3+}$$

### Content of kit

The kit contains two sets of reagents: one set is labeled in blue color (1 to 3) for iodine detection and the other set is labeled in brick color (A to C) for iron detection. The reagents are prepared in double-distilled deionized water (DDW). A total of 10 ml of the reagents provided in each bottle of kit are sufficient for testing 50–60 samples (a minimum of 50 samples is guaranteed). The iodine detection set (with blue color reagent bottle cap tops) contains (i) solution 1: 1M H_2_SO_4_ in DDW, (ii) solution 2: pre-calibrated KI solution (W/V) in DDW, and (iii) solution 3: acidified starch solution with preservative. The iron detection set (with red color reagent bottle cap tops) contains (a) solution A (1N HCl), (b) solution B (pre-calibrated KSCN), and (c) solution C (pre-calibrated H_2_O_2_). The kit also provides a white acrylic test plate with a dark cardboard cover plate and a set of 5 toothpicks as well as a specific volume spoon-1 (specially designed blue color spoon to give approximately 1 g of sample). Apart from these, the kit also contains one vial of salt free of iron and iodine, to be used as a negative control, and one vial of salt containing iron and iodine (cap top with half blue and red color), to be used as a positive control. This allows for validation of the kit, and it should be used only once at the beginning to check the same. Finally, it also provides an “Image strip” that depicts iron/iodine positive or negative color development. Furthermore, solution C will be packaged in a dark color bottle keeping in view the susceptibility of H_2_O_2_ to light.

### Procedure: (Also refer to the video)

**FIGURE 1:**
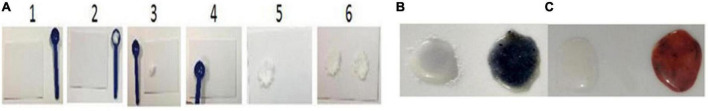
Preparation of samples in steps (1-6) for the detection of iodine and iron **(A)** and validation of reagents for the analysis of iodine and iron, and sample preparation – Non-development and color development demonstrate the presence of iodine, and salt Iodine samples (−) and (+) (**B**, blue), and iron and salt Iron samples (−) and (+) (**C**, violet).

Preparation of samples for validation of reagents for iodine testing (Figure 1A):

*Step-1* Keep the acrylic test plate and spoon on a table or any plane surface.

*Step-2* Take half a spoon of salt from both negative and positive control vials (negative first followed by positive).

*Step-3* Place them on the plate to form two mounds. Wipe clean the spoon with tissue paper.

*Step-4* Press with the spoon’s bottom to make wells. Wipe clean the spoon with tissue paper.

Now you are ready for validation with the control salt samples provided with the kit.

*Step-5* Add 2 drops of each reagent 1, 2, and 3, on the first sample, then wait for 2–3 min, and mix with a toothpick. Nondevelopment of color indicates the absence of iodine in the negative control sample, and the development of dark violet bordering to gray color with a yellowish outer-circle tinge in positive control indicates the presence of iodine (Figure 1B).

*Important:* Rinse the plate with water and wipe clean it with tissue paper for iron validation testing or turn the plate around if water is not available.

*Repeat steps 1-4 for the preparation of samples for validation of reagents for iron testing.*

*Step-6* Add 2 drops of each reagent A, B, and C, on both negative and positive control samples, wait for 30 s, and mix with a toothpick. Nondevelopment of color indicates the absence of iron in the negative control sample, and the development of brick red color in positive control after 30 s indicates the presence of iron (Figure 1B).

The proper performance of steps 5 and 6 indicates that the reagents are working satisfactorily.

(1). *(Kindly note that there may be some faint color development as theoretically no salt is totally devoid of iodine and iron that may be present as minor contaminants.).*
(2). Testing DFS for the presence of iron and iodine.
(3). Repeat the procedure outlined in steps 1-4 with the DFS test sample. Make 2 spots of DFS (one for iodine and the other for iron). Add reagents in the sequence mentioned in step 5 for iodine testing and follow the reagent addition sequence mentioned in step 6 for iron testing.

*Important Note:* Remember that added iron exists in many chemical forms like ferric sodium EDTA, also known as sodium ferric ethylenediaminetetraacetate (NaFe EDTA), free iron, iron sulfate, and iron fumarate. While testing iron in the DFS sample, you should first add only solutions A and B. The development of brick color indicates the presence of iron in oxidized form. In case, the brick red color does not develop on the addition of reagents A and B, then only add solution C. The development of deep brick color in the sample indicates the presence of iron in reduced form (Figures 1B, C*).*

The efficacy of the developed kit was tested under field conditions, to test the ability of the kit to distinguish between the households using either DFS or only iodized salt. The details of the methodology and results are given in the published manuscript (27).

## Results

**FIGURE 2:**
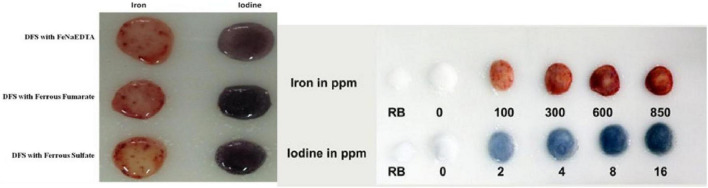
The kit successfully detects iron from a variety of fortificants as well as iodine in DFS and provides a color gradation chart.

The results in Figure 2 indicate that various forms of iron used for the making of DFS are successfully detected by kit reagents. Furthermore, experience in the laboratory and field shows that the kit allows to detect both iodines at as low as 5 parts per million (ppm) and as high as 40 ppm and iron levels as low as 100 parts per million (ppm) and as high as 1,000 ppm, in salt containing iron in various compounds used for fortification (ferrous as well as ferric). The brick red color can differentiate iron levels of 50, 100, 500, 800, and 1,000 ppm (Figure 2). The iron levels obtained by the kit were inspected by two independent observers of DFS samples and showed a good correlation. However, one of the limitations of the kit remains the inconsistency in the reproducibility of color charts. More efforts are needed for further improvisation. Finally, the results obtained for household salt samples collected from DFS intervention and control districts were very valuable to analyze how successful the DFS offtake from fair price shops in the targeted populations (27). In this regard, 100% specificity for iron was highly helpful (27).

The set of reagents A–C and 1–3 can be used to test about 50 salt samples. The kit is packed in an adequate size convenient for a person to carry to the field. The reagents in the kit are provided in a ready-to-use form as deionized water may not be available in the field.

We, therefore, developed a kit that allows the simultaneous qualitative identification of both iodine and iron in DFS, with properties like (a) two separate reaction matrices producing different color endpoints, (b) a combination of acidic medium and an oxidizing agent capable of de-encapsulation, (c) ensuring the conversion of ferrous to the ferric form of all the possible iron fortificants in use, and (d) ease of use under field conditions to enable the end-users such as retailers, Anganwadi workers, and households.

Furthermore, a graded color chart provided with the kit also helps to estimate the semiquantitative levels of iodine as well as iron. Finally, the iron detection kit components can be separately used for only iron detection in DFS. The stability of reagents has been tested for 6 months.

This research work applies a combination of radicals from hydrogen peroxide, with an organic solvent as a chemical pretreatment method, to disrupt the cell wall of microalgae and simultaneously extract lipids from the biomass in a one-step biphasic solution (24–26). The profound hybrid biphasic system constituting hydrogen peroxide and water (24–26) shows a great potential to radically disrupt the encapsulating lipids in a single-step approach that allows the detection of released iron through conversion to an oxidized form amenable to potassium thiocyanate reaction.

The present kit scores over the kit developed at the NIN, Hyderabad, for the simultaneous double fortification of edible salt with iron and iodine (20, 21). The attached video shows the ease of use of the MUNNG Optima kit (Supplementary material). The kit may also be used to detect the presence of iron in double-fortified salt, and the presence of iodine can be assessed by using the improvised iodized salt kit reported earlier (19, 21). One limitation of our kit is the quality and size of their color charts. Rather than images representative of the granular, irregular surface of actual samples, the charts show clear, homogeneous colored discs, which are not well related to the actual appearance of salt. The gradation chart requires further improvisation in terms of consistency.

## Discussion

The kit allows the detection of both iron and iodine in DFS prepared using ferrous sulfate, encapsulated ferrous fumarate, and NaFe EDTA salt as a premix. Results prove the ability of kit reagents to detect salt iodine content albeit in a delayed manner. It indicates some kind of interference in the iodine starch reaction from the iron compounds-rich milieu. The proof of concept about the utility of the developed kit has been established under real-life conditions during a survey conducted to assess iodine status in DFS-supplied districts and DFS non-supply districts (27). The ability of the kit to distinguish the households using either DFS or iodized-only salt was used to assess the iodine status of women in the reproductive age group and children (27). The major drawbacks of these techniques are that they are valid for a limited range of concentrations and are not very precise. However, these qualitative detections are advantageous because they are simple, rapid, and quite easy to perform. Most importantly, the increasing demand for DFS and its wide distribution presents the right time for the introduction and utilization of this field-testing kit for monitoring DFS. The fortification of iodized salt with iron compounds is likely to be the most widely used strategy to control and eliminate iron deficiency anemia. However, to be fully effective in correcting both iodine and iron deficiency, DFS must not only reach the entire affected population (particularly, the groups that are the most susceptible including pregnant women and young children), but iodized salt also needs to be adequately fortified with iron compounds. Hence, the FSSAI guidelines emphasize process indicators, in particular, those related to the monitoring of DFS at the production and/or importation levels, and DFS use in the population. Such monitoring necessarily involves both governments and the salt industry, with close collaboration between the public and private sectors. The process indicators refer to the indicators that monitor and evaluate the salt iodization process. These indicators reflect the salt iodine content at the production/importation site and at the household level, and in some instances, checking at the retail/wholesale level. The assessment of iodized salt being used in the food industry must also be considered. The actual availability of iron and iodine from DFS, at the consumer level, may vary over a wide range as a result of (i) variability in the amount of iron/iodine added during the iodization process; (ii) uneven distribution of iron/iodine in the DFS, within batches and individual bags, due to insufficient mixing of salt after the salt iodization process and/or variation in particle size of salt crystals in a batch or bag; (iii) the extent of loss of iron/iodine due to salt impurities, packaging (for instance, 1 kg vs. 20 or 50 kg), and environmental conditions during storage and distribution; (iv) loss of iron/iodine due to food processing, and washing and cooking processes in the household; (v) the availability of non-iodized and iron-fortified salt from unconventional marketing sources.

The recent evaluations of these kits for salt iodine suggest that the color reaction is not a suitable quantitative indicator of the iodine content (28–30). The same is also true for the present kit as well. These kits should, therefore, be regarded as qualitative rather than quantitative and are most appropriate to indicate the presence or absence of iodine but do not indicate the concentration. An advantage of the rapid test kits is that they may be used in the field to give a rapid result. Therefore, they are useful to health workers and others who are involved in carrying out spot checks on food quality or household surveys. They may also play a valuable educational role; in that, they provide a visible indication that salt is iodized. Accordingly, they may be used for demonstration purposes in schools and other institutions. The attached video is self-explanatory and demonstrates the simplicity and ease of using the MUNNG Optima kit. However, as the rapid test kits do not give a reliable estimate of iodine content (28–30), these results must be backed up by titration. All the considerations listed above for iodized salt are equally applicable to DFS. Thus, the above kit may be a valuable tool for the quality assessment of DFS that needs further validation and scientific scrutiny. One such scientific assessment undertaken in a blinded fashion to us with statistically valid samples analyzed by both kit and laboratory methods supports the usefulness of the kit for rapid check of iodine and iron in DFS samples (unpublished observations). The present price of the kit is INR 250.00 for 50 estimates and is comparable to different kits in the market that estimate iron and iodine separately. With large demand, a price reduction to INR 150 is possible.

## Conclusion

The kit reagent combined allows for rapid detection of iron and iodine in DFS. Iron detection is ensured in various iron-containing fortificant premixes. The provision of diluted H_2_O_2_ ensures the presence of oxygen-free radicals that enhances iodine release that is captured by concentrated KI making iodine detection an easy task. The kit needs further scientific proving.

## Data availability statement

The raw data supporting the conclusions of this article will be made available by the authors, without undue reservation.

## Author contributions

UG: investigation conducting a research and investigation process, specifically performing the experiments, or data/evidence collection, validation verification, whether as a part of the activity or separate, of the overall replication/reproducibility of results/experiments and other research outputs, and acquisition of the financial support for the project leading to this publication. DG: conceptualization ideas, formulation or evolution of overarching research goals and aims, methodology development or design of methodology, and formal analysis application of statistical techniques. NG: collecting and supervising salt samples detection of iodine and iron from household samples, undertaken video filming, editing and commentary. MG: conceptualization- ideas, formulation or evolution of overarching research goals and aims, supervision oversight and leadership responsibility for the research activity planning and execution, including mentorship external to the core team, and writing—original draft preparation, creation and/or presentation of the published work, specifically writing the initial draft (including substantive translation). All authors contributed to the article and approved the submitted version.
